# Priming of Plant Defenses against *Ophiostoma novo-ulmi* by Elm (*Ulmus minor* Mill.) Fungal Endophytes

**DOI:** 10.3390/jof7090687

**Published:** 2021-08-25

**Authors:** Clara Martínez-Arias, Juan Sobrino-Plata, Luis Gil, Jesús Rodríguez-Calcerrada, Juan Antonio Martín

**Affiliations:** Departamento de Sistemas y Recursos Naturales, ETSI Montes, Forestal y del Medio Natural, Universidad Politécnica de Madrid, 28040 Madrid, Spain; juan.sobrino@upm.es (J.S.-P.); luis.gil@upm.es (L.G.); jesus.rcalcerrada@upm.es (J.R.-C.); juan.martin.garcia@upm.es (J.A.M.)

**Keywords:** fungal endophytes, Dutch elm disease, defense, gene expression, priming

## Abstract

Some fungal endophytes of forest trees are recognized as beneficial symbionts against stresses. In previous works, two elm endophytes from the classes Cystobasidiomycetes and Eurotiomycetes promoted host resistance to abiotic stress, and another elm endophyte from Dothideomycetes enhanced host resistance to Dutch elm disease (DED). Here, we hypothesize that the combined effect of these endophytes activate the plant immune and/or antioxidant system, leading to a defense priming and/or increased oxidative protection when exposed to the DED pathogen *Ophiostoma novo-ulmi*. To test this hypothesis, the short-term defense gene activation and antioxidant response were evaluated in DED-susceptible (MDV1) and DED-resistant (VAD2 and MDV2.3) *Ulmus minor* genotypes inoculated with *O. novo-ulmi*, as well as two weeks earlier with a mixture of the above-mentioned endophytes. Endophyte inoculation induced a generalized transient defense activation mediated primarily by salicylic acid (SA). Subsequent pathogen inoculation resulted in a primed defense response of variable intensity among genotypes. Genotypes MDV1 and VAD2 displayed a defense priming driven by SA, jasmonic acid (JA), and ethylene (ET), causing a reduced pathogen spread in MDV1. Meanwhile, the genotype MDV2.3 showed lower defense priming but a stronger and earlier antioxidant response. The defense priming stimulated by elm fungal endophytes broadens our current knowledge of the ecological functions of endophytic fungi in forest trees and opens new prospects for their use in the biocontrol of plant diseases.

## 1. Introduction

Forest trees are exposed to biotic disturbances, which in some cases can prompt the rapid decline or even near extinction of some species [1,2]. Constant pathogen pressure has led plants to develop sophisticated adaptations to mount immune responses and trigger complex defense networks to counteract the deleterious effect of pathogens [3,4]. When a pathogen attack is initiated, the plant recognizes pathogen-released molecules (pathogen or microbe-associated molecular patterns; PAMPs or MAMPs) through plant receptors (pattern-recognition receptors, PRR), inducing the so-called PAMP or MAMP-triggered immunity (PTI or MTI) [5]. This PTI together with the recognition of effector molecules released by the pathogen (effector triggered immunity, ETI) leads to the local accumulation of signaling molecules such as phytohormones salicylic acid (SA), jasmonic acid (JA), or ethylene (ET) that trigger the plant immune signaling network [6]. SA-signaling is usually activated against biotrophic pathogens (i.e., those living at the expense of live host cells), while JA-signaling activates in response to necrotrophic pathogens (i.e., those that kill the living cells to feed on them) [7]. Nevertheless, the antagonism or synergy between SA and JA, and between any of them and other hormones such as abscisic acid or ET, will determine the specific nature of the response in each case [8]. The plant defense response culminates with the synthesis of a chemical arsenal with effective antimicrobial properties, such as phytoalexins or chitinases. The activation of the plant oxidative machinery is another early response activated within minutes from the pathogen recognition, producing reactive oxygen species (ROS) that can act directly against the microorganism or serve as signals to activate a systemic defense response [9]. Nevertheless, ROS production needs to be controlled to non-toxic levels by activating ROS-scavenging mechanisms [10]. Increasing evidence demonstrates that the host RNA interference machinery is also involved in the modulation of the plant immunity, including pathogen perception, ROS production, signal transduction, or downstream immune response [11]. Furthermore, the trafficking of small-RNA between the plant and the pathogen has been recently discovered to modulate the virulence of the pathogen and the expression of plant genes, respectively [12].

Intimate associations with beneficial microorganisms can contribute to pathogen tolerance in plants [13,14,15]. Mycorrhizal fungi or plant-growth promoting rhizobacteria are perhaps the best-known microorganisms with proven implications on plant nutrient uptake and plant growth [16]. Although the ecological functions of the vast majority of microbes making up the plant microbiome remain largely unexplored [17], growing evidence points towards a role of some endophytes in maintaining plant fitness against biotic and abiotic stresses [18]. Fungal endophytes can establish direct interactions with pathogens (i.e., antibiosis, mycoparasitism, and niche competition) that lead to enhanced host resistance [19]. Indirectly, endophytes may support plant growth and physiology by improving nutrient acquisition or phytohormone synthesis, helping to counteract the negative effects of pathogen invasion [20]. Moreover, some endophytes have been reported to stimulate the plant immune system, helping to fight more efficiently against broad spectrum pathogens [21].

Certain symbionts enhance the plant defense by increasing the antioxidant state [22]. A light ROS burst induced by endophytic colonization activates the plant antioxidant machinery, enhancing the ROS-scavenging capacity at the moment of pathogen attacks [23]. Beneficial symbionts can also reprogram the expression of plant defense genes via induced systemic resistance (ISR) [24]. The mechanism of ISR is similar to the pathogen-induced systemic acquired resistance (SAR) (i.e., activated through MAMPs recognition by PRR) but generally is regulated in a SA-independent manner, as JA and ET are the central regulating hormones [24]. Nevertheless, ISR regulation has also been reported to occur in a SA-dependent manner [25,26,27]. ISR prepares the plant to better combat pathogen attack in a process known as defense priming. The resulting primed plant activates a faster, stronger, and more prolonged defense transcriptional response when attacked by a pathogen than non-primed plants [28,29,30]. However, the extent to which fungal endophytes participate in defense reprogramming remains largely unexplored. Most of the existing knowledge supporting the role of fungal endophytes in biotic stress tolerance in forest trees is based on evaluations of external symptoms [31,32,33,34,35], with some but still scarce evidence linking enhanced stress tolerance to the modulation of the plant’s defense gene expression caused by the symbiont [36,37].

In the present work, we aim to deepen the knowledge regarding the role of fungal endophytes of forest trees as inducers of defense mechanisms against pathogens by exploring the Dutch elm disease (DED) pathosystem. DED is a vascular wilt disease that has caused massive deaths of *Ulmus* species populations in Europe and North America in the last 100 years [38,39,40]. The causal agents are several species in the genus *Ophiostoma*, being *O. novo-ulmi* Brasier responsible of the current pandemic [41,42]. In the last years, the discovery of elm fungal endophytes with potential antagonism to *O. novo-ulmi* has been object of research [43,44,45]. In this way, a recent metabarcoding study performed on *U. minor* genotypes with varying levels of susceptibility to DED (ranging from tolerant to susceptible) revealed that the relative abundance of some fungal core endophytes was associated to host resistance [46]. More recently, two fungal isolates belonging to this group of putative beneficial endophytes (classes Cystobasidiomycetes and Eurotiomycetes), and an additional isolate (Phaeosphaeriaceae) with a strong in vitro antibiotic activity towards *O. novo-ulmi*, have shown positive effects on plant stress tolerance [47,48]. We hypothesize that (i) *U. minor* PRRs recognize the MAMPs secreted by these endophytic fungi, triggering a signaling cascade that primes plant defense when the plant is exposed to *O. novo-ulmi*; (ii) endophyte presence in *U. minor* tissues modifies the plant antioxidant machinery, enhancing protection against the oxidative stress caused by the pathogen; and (iii) both previous mechanisms reduce the spread and negative effects of *O. novo-ulmi* in host plants. To test these hypotheses, a mixture of the three fungal endophytes mentioned above were inoculated into one-year-old *U. minor* plants and subsequently inoculated with *O. novo-ulmi*. To evaluate if the DED resistance level has any influence in the priming of defense mechanisms, we used two DED-resistant genotypes and one DED-susceptible genotype. The short-term defense and antioxidant response was evaluated in these plants using JA/ET and SA-responsive marker genes taken from the published *U. minor* transcriptome [49], in addition to quantifying two key enzymes involved in the plant antioxidant system. This response was compared with that produced by the single inoculation of *O. novo-ulmi*.

## 2. Materials and Methods

### 2.1. Plant and Fungal Material

Three *U. minor* genotypes from Spain with contrasting levels of susceptibility to DED were propagated through aerial cuttings. The susceptibility to DED was previously evaluated in controlled inoculations with *O. novo-ulmi* according to the protocol followed by the Spanish elm breeding program [50]. The MDV1 genotype (from Madrid) was classified as susceptible (>75% of foliage wilting 60 days after *O. novo-ulmi* inoculation), while MDV2.3 (from Madrid) and VAD2 (from Valencia) were both classified as resistant (<15% of foliage wilting).

For aerial cutting production, annual shoots from adult trees growing in a conservation plot at the “Puerta de Hierro” Forest Breeding Center (Madrid, Spain) were harvested at the end of January 2019. Shoots were immediately cut to 15 cm-long segments containing 4–6 buds. Then, the proximal ends of the cuttings were immersed (2 cm) in a solution of 0.6% auxin-like growth-regulating hormone (indole-3-butyric acid) for 10 s. After being washed with water, the cuttings were planted in 5 dm^3^ pots filled with a mixture of peat, sand, and vermiculite (1:1:1 in volume proportions) and kept in a greenhouse.

Three fungal endophytes identified as YM11, P5, and YCB36 isolated from elm twigs in 2014 and 2015 were selected for this experiment [44]. YM11 is a dark septate endophyte within order Chaetothyriales (Eurotiomycetes), P5 is a yeast assigned to the genus *Cystobasidium* (Cystobasidiomycetes), and YCB36 is a filamentous fungus of the Phaeosphaeriaceae family (Dothydeomycetes), which was tentatively assigned to the genus *Hydeomyces* [47]; these strains are deposited in the Spanish Type Culture Collection (CECT) under the codes CECT 13193, CECT 13192, and CECT 21178, respectively. P5 and YM11 endophytes are within the core taxa found in higher proportions in DED-resistant *U. minor* trees [46], while YCB36 was selected because of its high antibiotic activity against *O. novo-ulmi* [47]. These endophytes are conserved in our lab immersed in an autoclaved solution of 15% glycerol at −80 °C.

Two months before the experiment, the three endophytic fungi were cultured in the dark on yeast extract agar (YEA) medium at 22 °C. Every 15 days the colonies were subcultured onto fresh YEA mediums. To obtain the endophyte inoculum, endophytes were grown individually on YEA medium with (YM11 and YCB36) or without (P5) an autoclaved cellophane layer for 10 days at 22 °C in the dark. P5 yeast cells were scraped from the agar using a sterile spatula and suspended in sterile distilled water. YM11 colonies, composed of a mixture of hyphal and budding cells, were disintegrated with an all-glass tissue homogenizer to obtain a homogenous cell suspension. YCB36 did not produce enough conidia and therefore spores were replaced by mycelial fragments obtained by using an all-glass tissue homogenizer. The resulting suspension was filtered using sterilized cotton wool to avoid large hyphal fragments and retain only the fragments of 1–4 cells. An aqueous suspension of a mixture of the three endophytes (as yeast cells or mycelial fragments) was adjusted to a final inoculum concentration of 5 × 10^6^ cells mL^−1^ using a hemocytometer.

The *O. novo-ulmi* subspecies *americana* isolate SOM-1 [51] was used for pathogen inoculation. Mycelial plugs of this isolate were cultured on malt extract agar (MEA) two months before the experiment, kept in the dark at 22 °C, and subcultured every 15 days on the same substrate. To obtain *O. novo-ulmi* spores, mycelial plugs were grown in Erlenmeyer flasks with Tchernoff’s liquid medium [52] under constant shaking at 22 °C to induce sporulation. Three days later, spores were collected by centrifugation and adjusted with sterile distilled water (dH_2_O) to 10^7^ blastospores mL^−1^ using a hemocytometer.

### 2.2. Experimental Design

**Figure 1:**
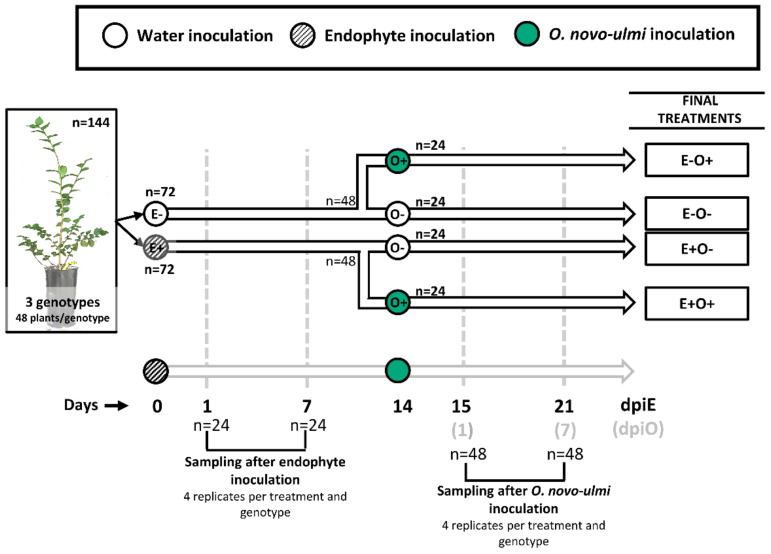
Diagram of the experimental design. Briefly, the experiment comprised 144 vegetative propagated plants, i.e., 48 clonal plants per genotype. At the beginning of the experiment, half of the plants (n = 72; 24 plants per genotype) were inoculated with 50 µL of a suspension of endophyte cells (E+) while the other half was inoculated with 50 µL of dH_2_O (E−). Four replicate plants per genotype and treatment were harvested for sampling at days 1 and 7 after endophyte inoculation (dpiE). At day 14, the non-harvested E− and E+ plants (n = 48 in each treatment, 16 plants per genotype) were divided in two groups: plants inoculated with *O. novo-ulmi* (n = 24) or with dH_2_O (n = 24). Thus, we applied four treatments: E−O− plants (mock-inoculated plants in both treatment dates), E−O+ plants (mock-inoculated plants subsequently inoculated with *O. novo-ulmi*), E+O− plants (endophyte-inoculated plants subsequently inoculated with water), and E+O+ plants (endophyte-inoculated plants subsequently inoculated with *O. novo-ulmi*). Four replicate plants per genotype and treatment were harvested for sampling at days 1 and 7 after *O. novo-ulmi* inoculation (dpiO: at days 15 and 21 of the experiment).

Plants were grown in 5 dm^3^ pots during the whole experiment under greenhouse conditions: 600 µmol m^−2^ s^−1^ maximum irradiance, 26/16 °C day/night temperature, and 50/60% day/night air relative humidity. All the plants were distributed in a single plot divided in six blocks. Each block included 2 replicate plants per genotype and treatment (see below), randomly positioned. In total, the experiment comprised 144 vegetative propagated plants with 48 replicates per genotype. Half of the plants (n = 72; 24 plants per genotype) were initially inoculated with 50 µL of the endophyte inoculum suspension (E+), which was delivered into the xylem tissues through a transverse cut made with a sharp blade at 5 cm from the substrate level, allowing the absorption of the inoculum [53]. The other half was inoculated with 50 µL of dH_2_O (E−) following the same procedure (Figure 1). Four replicate plants per genotype and treatment were sampled at 1 and 7 days post endophyte inoculation (dpiE). In each sampling day, a 10 cm-long stem piece was cut, including the inoculation wound in the middle point. The stem piece was immediately frozen in liquid N_2_ and stored at −80 °C until further use. Fourteen days later, the non-harvested E− and E+ plants (n = 48 in each treatment, 16 plants per genotype) were divided to be inoculated either with *O. novo-ulmi* (n = 24) or dH_2_O (n = 24). *O. novo-ulmi* inoculations were performed 2 cm below the wound performed for endophyte inoculation and in the opposite side of the stem. Fifty μL of the *O. novo-ulmi* spore suspension or dH_2_O were delivered into the xylem following the same procedure described above. In summary, we applied four inoculation treatments: E−O− (mock-inoculated plants in both treatment dates), E−O+ (mock-inoculated plants subsequently inoculated with *O. novo-ulmi*), E+O− (endophyte-inoculated plants subsequently inoculated with water), and E+O+ (endophyte-inoculated plants subsequently inoculated with *O. novo-ulmi*) (Figure 1). Four replicate plants per genotype and treatment were sampled at 1 and 7 days post *O. novo-ulmi* inoculation (dpiO; i.e., at days 15 and 21 from the initial endophyte inoculation). In each sampling day, we cut a 10 cm-long basal stem piece including both endophyte and *O. novo-ulmi* inoculation wounds to study local responses. In addition to the basal stem piece, in the plants harvested at day 21 from the initial endophyte inoculation, a 15 cm-long apical stem piece and a 10 cm-long intermediate stem piece were cut to study pathogen dispersal. Endophyte presence was only assessed locally. The 10 cm-long intermediate fragment was selected by measuring the plant height and cutting 5 cm above and below the middle point, and the distal stem fragment by cutting the last 15 cm of the stem. In all cases, the stem pieces were immediately frozen in liquid N_2_ and stored at −80 °C until further analyses.

### 2.3. O. novo-ulmi Impact and Spread in Inoculated Plants

We estimated the negative effect of *O. novo-ulmi* in inoculated plants and the pathogen presence in xylem tissues. First, several physiological traits were assessed to detect any dysfunction caused by the pathogen [54]. Second, wood discoloration (browning of vascular tissues) associated to the accumulation of phenolic compounds, gums, and tyloses [55,56] was measured as a proxy of the plant reaction to the pathogen. Third, we quantified the presence of *O. novo-ulmi* in local, intermediate, and distal stem fragments through real-time PCR (see next section). Foliar wilting was not considered to evaluate the impact of the disease, as it is not a reliable trait when using one-year-old plants [53].

Plant physiology was assessed before the plant harvest at day 21. Leaf gas exchange was measured in an intact leaf in each plant replicate per treatment (n = 4) with a LI-6400XT portable system (LiCor Inc., Lincoln, NE, USA) using a 2 cm^2^ chamber with a LED light source. Chamber conditions were adjusted to 1000 µmol m^−2^ s^−1^ photon flux density (PFD), 400 ppm air CO_2_ concentration, and 25 °C air temperature. Net photosynthesis (P_n_), stomatal conductance (g_s_), intercellular concentration of CO_2_ (C_i_), and the transpiration rate (E) were recorded.

Wood discoloration was measured in the intermediate stem fragments. A transverse cut was made in the middle of the stem segment with a sharp blade and then was observed and photographed with a Leica S9i stereomicroscope (Leica Microsystems GmbH, Wetzlar, Germany). The area of the browning tissue in the xylem transverse section was measured using the ImageJ software http://imagej.nih.gov/ij/ (accessed on 8 December 2020).

### 2.4. Pathogen and Endophyte Quantification in Plant Tissues

To compare the extent of the pathogen spread among treatments, *O. novo-ulmi* presence was quantified in the local, intermediate, and distal stem pieces from plants harvested at 21 dpiE. Only plants from treatments including the *O. novo-ulmi* inoculation (i.e., E−O+ and E+O+) were analyzed. To evaluate the effectiveness of the endophyte inoculation, the presence of the three inoculated endophytes was quantified in the local stem fragment from plants harvested at 21 dpiE. In this case, plants from treatments E+O−, E+O+, and E−O− were used. In all cases, the stem material was ground to a fine powder using a ball mill (Mixer mill MM 400, Retsch GmbH, Haan, Germany) and DNA was extracted using the plant/fungi DNA isolation kit from Norgen (Norgen Biotek Corp., Thorold, Ontario Canada). Primer sequences were designed within the Sanger sequenced ITS region of ribosomal DNA from *O. novo-ulmi*, P5, YM11, and YCB36 using Primer3 Version 0.4.0 http://bioinfo.ut.ee/primer3-0.4.0/primer3/ (accessed on 8 April 2020). The primer pairs were set as described in Table S1. Fungal DNA was standardized per quantity of plant DNA by amplifying a fragment of the *U. minor* ITS region (ITS-Ulmi) (forward primer: 5′-ATATGTCAAAACGACTCTCGGCAAC-3′/reverse primer: 5′-AACTTGCGTTCAAAGACTCGATGGT-3′). Quantifications of fungal DNA were performed in triplicates using SSoFast EvaGreen^®^ Supermix (Bio-rad laboratories, Hercules, California, USA) in a CFX96 real-time PCR detection system thermocycler (Bio-rad laboratories, Hercules, California, USA) with a standard amplification protocol. Each quantification plate contained a standard curve with *O. novo-ulmi*, P5, YM11, or YCB36 DNA for its corresponding ITS primer pair (0.5, 0.05, 0.005, and 0.0005 ng of fungal DNA), and another curve with plant DNA for the ITS-Ulmi primer pair (50, 10, 2, and 0.4 ng of plant DNA). The logarithm of the starting quantity of DNA and the value of the cycle threshold (Ct) obtained for each DNA quantity had a linear relationship that allowed us to obtain a regression equation between both variables for plant and fungal DNA. Moreover, each plate contained two replicates of each sample: one for the amplification of the specific fungal ITS and the other one for the amplification of the ITS-Ulmi. Therefore, the Ct value obtained in each sample allowed us to determine the quantity of fungal DNA and plant DNA. The results of the quantification were expressed as a quantity of *O. novo-ulmi* or endophyte DNA (ng) relative to quantity of plant DNA (ng).

### 2.5. Gene-Expression Analysis

The local stem samples harvested at 1, 7, 15, and 21 dpiE (including treatments E−O− and E+O− at days 1 and 7, and treatments E−O−, E+O−, E−O+, and E+O+ at days 15 and 21; see Figure 1) were used to evaluate the expression of defense genes after endophyte and *O. novo-ulmi* inoculations. Three biological replicates per treatment were analyzed and 11 genes associated with JA/ET and SA signaling networks were selected. For gene selection, we reviewed literature regarding SAR and ISR studies on model plants [6,24,29] and sought homologue genes in *U. minor* transcriptome databases [49,57] (Table S2). Regarding SA-biosynthesis, we selected the *PAL1* and *SARD1* genes as representatives of the phenylalanine ammonia-lyase and the isochorismate pathways, main paths for SA-biosynthesis [58,59]. Among the SA-responsive genes, we selected the *PR4* gene, which encodes a chitinase protein, and the *WRKY70* transcription factor, implicated in feedback biosynthesis of SA [60,61]. Moreover, five genes involved in JA/ET signaling were selected. The *OPR3* gene was chosen for encoding one of the key enzymes involved in JA-synthesis [62], as well as the genes *MYC2* and *EIN3* for being regulators of JA and ET responsive genes [63,64], respectively. Additionally, we selected the *THI2-like* gene that encodes a JA-induced thionin with antimicrobial properties [65] and the *bHLH13* gene for being a *MYC2* repressor [66]. Finally, we chose the transmembrane *PIP2* gene involved in the microbial recognition and elicitation of the immune response [67].

For gene expression analyses, frozen stem fragments were ground to a fine powder using a ball mill (Mixer mill MM 400, Retsch GmbH, Haan, Germany) and the RNA was extracted using the plant RNA isolation kit from Norgen (Norgen Biotek Corp., Thorold, Ontario, Canada). First-strand cDNA was synthesized from 1 µg total RNA from each sample using PowerScriptIII reverse transcriptase (Invitrogen, Waltham, Massachusetts, USA) according to the manufacturer’s instructions. Primer pairs were designed according to the sequences identified in *U. minor* transcriptomes (Table S2). Quantitative RT-PCRs were performed in triplicates using the SSoFast EvaGreen^®^ Supermix (Bio-rad laboratories, Hercules, California, USA) in a CFX96 real-time PCR detection system thermocycler (Bio-rad laboratories, Hercules, California, USA) with a standard thermal profile. Three technical replicates were processed for each PCR run. To compare the data from different PCR runs or cDNA samples, the mean of the threshold cycle (Ct) values of the three technical replicates was normalized to the mean Ct value of Ri18S (18S ribosomal RNA, housekeeping gene from *U. minor*), which showed consistent and constant expression in previous works [48,57]. The ΔΔCt method was used to obtain expression ratios [68].

### 2.6. Antioxidant Enzyme Activities

Protein extracts were prepared from the frozen powder material from all the local stem samples (harvested at days 1 and 7 dpiE, and 1 and 7 dpiO). The powder material of each biological replicate was pooled per genotype and treatment. Proteins were extracted from 0.3 g of the pooled powdered material by using 1 mL of extraction buffer solution. This solution was prepared as described by Martínez-Arias et al. [48].

Ascorbate peroxidase (APX) and glutathione reductase (GR) activities were determined in gel after the separation of protein extracts by non-denaturing electrophoresis in polyacrylamide gels, following the protocols described by Sobrino-Plata et al. [69]. Protein loading for APX and GR detection was 15 µg. Antioxidant activities were quantified by taking images of acrylamide gels with the GelDoc^TM^ XR+ system (BioRad, laboratories, Hercules, California, USA) and the intensity of the detected bands was measured using the Image Lab software (BioRad laboratories, Irvine, California, USA). Enzymatic activities were expressed as values relative to the intensity of control samples. A representative gel from three independent assays per activity was presented (Figure S4). Relevant changes were established as those with a ±30% fold change relative to the control treatment (E− at 1 and 7 dpiE, or E−O− at 1 or 7 dpiO).

### 2.7. Statistical Analysis

We evaluated the effects of the factors ‘genotype’ (MDV1, MDV2.3, and VAD2) and ‘treatment’ (E−O−, E+O−, E−O+, and E+O+) on the different variables measured. Thus, gas exchange variables (P_n_, g_s_, Ci, and E) were analyzed by two-way analysis of variance (ANOVA) including main and interaction effects of factors ‘genotype’ and ‘treatment’ in the ANOVA model. Pathogen quantity and vascular browning on E−O+ and E+O+ samples were also analyzed by two-way ANOVA. Pathogen quantity was analyzed independently for local, intermediate, and distal stem fragments. The mean values were compared between genotypes and treatments with Tukey’s HSD post-hoc test (*p* < 0.05). For both variables, plants that were not inoculated with the pathogen (E+O− and E−O−) were discarded from the analysis due to the absence of vascular browning development.

Quantification of the endophyte presence was analyzed in endophyte-treated plants (E+O− and E+O+) and in control plants (E−O−). The results were analyzed independently for each endophyte by two-way ANOVA. The mean values were compared between genotypes and treatments with Tukey’s HSD post-hoc test (*p* < 0.05).

The analysis of gene expression was performed independently for each genotype and sampling date. The fold-change expression values in each sample date were obtained in relation to the mean value of the mock-inoculated treatment (E−O−). One-way ANOVAs were performed to evaluate the effect of the treatment in each sample date and genotype. On days 15 and 21 of the experiment (i.e., 1 and 7 dpiO), Tukey’s post-hoc analysis (*p* < 0.05) was performed to compare the four treatment groups.

When needed, data were log or square-root transformed prior to analysis to comply with normality and homoscedasticity assumptions. Statistical analyses were performed using STATISTICA version 8.0 (StatSoft, Tulsa, OK, USA).

## 3. Results

### 3.1. Plant Phenotypic Traits

The leaf gas exchange parameters were not affected by the inoculation of endophytes or *O. novo-ulmi* in any elm genotype (*p* < 0.05; Figure S1).

**Figure 2:**
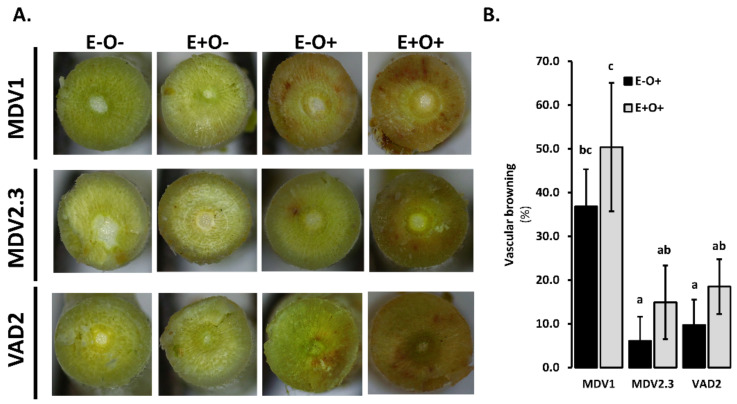
Vascular browning in stem transverse sections collected at day 21 of the experiment. (**A**) Representative picture of vascular browning in E−O− plants (mock-inoculated plants in both treatment dates), E−O+ plants (mock-inoculated plants subsequently inoculated with *O. novo-ulmi*), E+O− plants (endophyte-inoculated plants subsequently inoculated with water), and E+O+ plants (endophyte-inoculated plants subsequently inoculated with *O. novo-ulmi*) in genotypes MDV1 (DED-susceptible), MDV2.3 (DED-resistant), and VAD2 (DED-resistant). (**B**) Mean (± standard error) percentage of vascular browning in treatments E−O+ and E+O+. Different letters indicate significant differences between treatments and genotypes according to Tukey’s HSD post-hoc test (*p* < 0.05). The results of the ANOVA analysis are detailed in Table S3.

Browning of vascular tissues was only detected as a reaction to *O. novo-ulmi* inoculation, but not in E−O− or E+O− plants (Figure 2A). Therefore, statistical analysis was performed to compare the percentage of xylem browning between E−O+ and E+O+ plants (Table S3). E+O+ plants showed higher percentages of xylem browning than E−O+ plants (*p* < 0.05; Table S3). Moreover, the DED-susceptible genotype MDV1 showed larger browning areas than DED-resistant genotypes MDV2.3 and VAD2 (*p* < 0.05, Figure 2B; Table S3).

### 3.2. Pathogen Quantification in Plant Tissues

**Figure 3:**
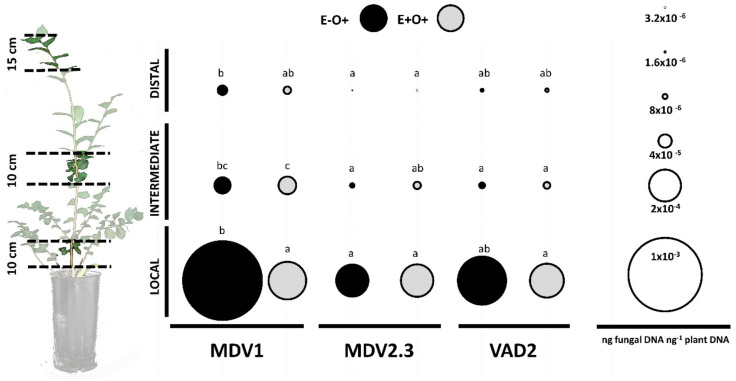
Presence of *O. novo-ulmi* at day 21 of the experiment in local (close to the inoculation point), intermediate, and distal stem pieces. Treatments E−O+ (mock-inoculated plants subsequently inoculated with *O. novo-ulmi*) and E+O+ (endophyte-inoculated plants subsequently inoculated with *O. novo-ulmi*) were evaluated in elm genotypes MDV1, MDV2.3, and VAD2. The mean value is represented by circles whose dimensions are proportional to the fungal DNA quantity (ng fungal DNA ng^−1^ plant DNA) as indicated in the circle scale. Different letters indicate significant differences between treatments and genotypes according to Tukey’s HSD post-hoc test (*p* < 0.05). Independent analyses were performed for local, intermediate, and distal stem fragments. The results of the ANOVA analysis are detailed in Table S3.

The *O. novo-ulmi* presence in stem fragments varied between *U. minor* genotypes at the end of the study (*p* < 0.05, Table S3). In plants not inoculated with the endophytes (E−O+), the susceptible genotype MDV1 showed a higher pathogen presence than the resistant genotype MDV2.3 in local, intermediate, and distal stem fragments, and also higher than the resistant genotype VAD2 in the intermediate stem fragment (Figure 3). Pathogen abundance in the stem was similar in MDV2.3 and VAD2 plants (*p* > 0.05, Figure 3).

The quantity of *O. novo-ulmi* in local stem tissues decreased in plants previously inoculated with endophytes (E+O+) in the MDV1 genotype (*p* < 0.05, Figure 3; Table S3).

### 3.3. Endophyte Quantification in Plant Tissues

**Figure 4:**
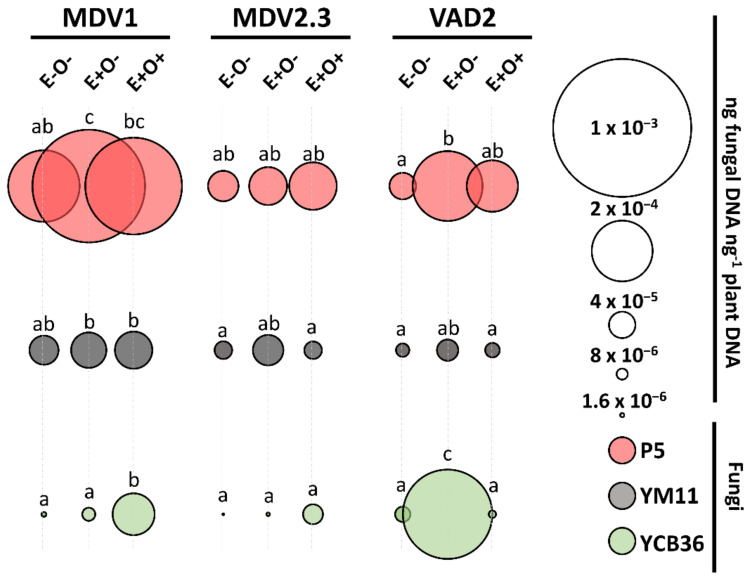
Presence of endophytes YM11 (gray circles), P5 (red circles), and YCB36 (green circles) in local stem pieces at day 21 of the experiment. The treatments E−O− (mock-inoculated plants in both treatment dates), E+O− (endophyte-inoculated plants subsequently inoculated with water), and E+O+ (endophyte-inoculated plants subsequently inoculated with *O. novo-ulmi*) were evaluated in the elm genotypes MDV1 (DED-susceptible), MDV2.3 (DED-resistant), and VAD2 (DED-resistant). The mean value in each case is represented by circles whose dimensions are proportional to the fungal DNA quantity (ng fungal DNA ng^−1^ plant DNA) as indicated in the circle scale. Different letters indicate significant differences between treatments and genotypes according to Tukey’s HSD post-hoc test (*p* < 0.05). Independent analyses were performed for each fungal strain. The results of the ANOVA analysis are detailed in Table S3.

The colonization of P5 and YM11 varied between genotypes, while YCB36 colonization was dependent on both the genotype and the treatment (i.e., *O. novo-ulmi* inoculation) (Table S3). None of the three inoculated endophytes significantly increased their presence in the genotype MDV2.3, while the presence of P5 and YCB36 endophytes tended to increase in genotypes MDV1 and VAD2 (Figure 4). YM11 presence did not significantly increase in any of the genotypes and treatments (E+O− and E+O+) compared to E−O− plants (Figure 4, Table S3). In the resistant genotype VAD2, both P5 and YCB36 presence increased in E+O− plants; nevertheless, YCB36 was significantly reduced after *O. novo-ulmi* inoculation (E+O+ plants). In the susceptible genotype MDV1, the presence of P5 increased in E+O− and E+O+ plants, while the presence of YCB36 only increased in E+O+ plants (Figure 4; Table S3).

### 3.4. Gene-Expression Analysis

#### 3.4.1. *U. minor* Response to *O. novo-ulmi* Inoculation

As expected, the response activated by *U. minor* against *O. novo-ulmi* depended mainly on the SA pathway. However, the induction level and its earlier or later expression varied among genotypes.

**Figure 5:**
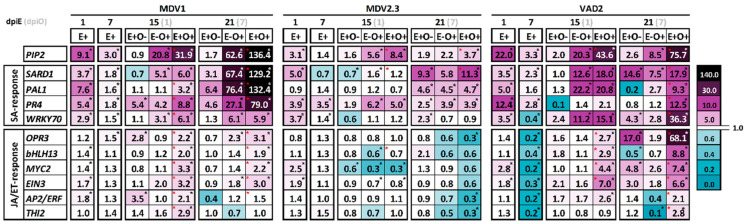
Quantitative reverse transcriptase polymerase chain reaction (qRT-PCR) results of salicylic acid (SA) and jasmonic acid/ethylene (JA/ET)-responsive genes at days 1 and 7 after endophyte inoculation (E+ treatment, dpiE), and at days 1 and 7 (days 15 and 21 of the experiment) after *O. novo-ulmi* inoculation (dpiO). Treatments are: E−O+ (mock-inoculated plants subsequently inoculated with *O. novo-ulmi*), E+O− (endophyte-inoculated plants subsequently mock-inoculated), and E+O+ (endophyte-inoculated plants subsequently inoculated with *O. novo-ulmi*). The values indicated in each box are the mean values of the fold-change expression with respect to the mean values of the mock-inoculated treatment (E− or E−O−) in each time point. Fold change values exhibiting statistically significant differences with respect to E− (at days 1 and 7 dpiE) or E−O− (at days 1 and 7 dpiO) are indicated with a black asterisk (or white for enhancing visualization) at the upper right corner of the box. The red asterisk indicated on the upper left side of the box indicates E+O+ fold change values exhibiting statistically significant differences with respect to E−O+ treatment. Further comparisons of Tukey’s HSD post-hoc test (*p* < 0.05) and means ± standard errors are indicated in Figures S2 and S3.

The susceptible MDV1 genotype showed a mild but significant induction of the SA-responsive genes *SARD1* and *WRKY70* (5.1- and 3.1-fold change expression values, respectively), and no induction of *PAL1* at 1 dpiO (see E−O+ treatment in Figure 5 and Figure S2). Nevertheless, a strong induction of the four SA-related genes was observed at 7 dpiO, with maximum induction levels of a 67.4- and 76.4-fold change for genes *SARD1* and *PAL1*, respectively. Moreover, some JA-related genes (*OPR3* and *EIN3*) were significantly activated at 7 dpiO (Figure 5).

Similarly, the VAD2 genotype also activated a SA-dependent response in reaction to *O. novo-ulmi* but with a different expression pattern than MDV1. Thus, a strong activation of genes *PIP2*, *SARD1*, *PAL1*, and *WRKY70* was detected at 1 dpiO, with fold changes ranging between 12.6 and 22.2. However, such activation was attenuated (*PIP2* and *SARD1*) or even reduced to basal levels (*PAL1* and *WRK70*) at 7 dpiO (Figure 5), in parallel to a repression of the JA/ET-responsive gene *THI2* (Figure 5). Moreover, in both MDV1 and VAD2, SA-related genes were induced in parallel with *PIP2* (Figure 5).

The resistant genotype MDV2.3 showed a different response than VAD2 and MDV1. This genotype also showed a SA-dependent response but with milder expression levels both at 1 and 7 dpiO (the expression levels barely exceeded a 6-fold change value). The *PIP2* gene was significantly upregulated at 1 dpiO but not at 7 dpiO, while *SARD1* and *PAL1* were induced with higher intensities at 7 dpiO. On the contrary, *PR4* showed a higher expression level at 1 dpiO than at 7 dpiO (Figure 5).

#### 3.4.2. *U. minor* Response to Endophyte Inoculation

As a general trend for all genotypes, modifications in SA and JA/ET-responsive genes were mainly produced at 1 dpiE and to a lesser extent at 7 dpiE. A relatively strong induction of SA-related genes was produced at 1 dpiE in all genotypes, with fold-change expression values ranging between 3.0 and 5.0, except for the *PAL1* gene in MDV1 which showed 7.6-fold change and the *PR4* gene in VAD2 with 12.4-fold change (Figure 5). At 7 dpiE, significant but milder gene overexpression was maintained in all the SA-responsive genes in MDV1 and VAD2, except for the *WRKY70* gene in VAD2 which showed a significant repression (Figure 5 and Figure S2). In the genotype MDV2.3, a significant enhancement of the *SARD1*, *PR4*, and *WRKY70* genes was detected at day 1, of which only *PR4* and *WRK70* continued to be overexpressed at day 7. On the contrary, *PAL1* was not overexpressed in MDV2.3 during these days (Figure 5 and Figure S2). As for the level of SA-related gene expression, the three genotypes showed higher induction of the *PIP2* gene at 1 than at 7 dpiE. MDV1 and VAD2 genotypes presented higher *PIP2* expression than MDV2.3 and significant overexpression of all SA-related genes (Figure 5). Moreover, the resistant VAD2 genotype showed the highest *PIP2* and *PR4* gene expression.

The expression of JA/ET-responsive genes was milder than SA genes, with expression patterns also variable among genotypes. In MDV1, slight but significant inductions were detected for *EIN3* and AP2/*ERF* at 1 dpiE, and for *OPR3* at 7dpiE (Figure 5 and Figure S3). In MDV2.3 and VAD2, only the *MYC2* and *EIN3* genes were significantly induced at 1 dpiE (Figure 5 and Figure S3). Remarkably, all the JA/ET-responsive genes in VAD2 were significantly repressed at 7 dpiE (Figure 5 and Figure S3).

Changes in the expression of some genes at 15 and 21 dpiE were also observed in endophyte-inoculated plants that were subsequently inoculated with water instead of *O. novo-ulmi* (E+O− treatments) (Figure 5). In the VAD2 genotype, *SARD1* and *OPR3* were the most strongly induced genes with fold change values of 14.6 and 17 at 21 dpiE, respectively. In MDV2.3, *SARD1* showed the highest overexpression with 9.3-fold change at 21dpiE (Figure 5 and Figure S2). In genotype MDV1, milder upregulation was produced in *PR4*, *OPR3*, and *AP2/ERF* at 15 dpiE (Figure 5 and Figures S2 and S3).

#### 3.4.3. *U. minor* Response to *O. novo-ulmi* in Plants Previously Inoculated with Endophytes

In general, endophyte-inoculated plants upregulated SA-responsive genes in response to *O. novo-ulmi* (E+O+ plants) to a higher extent than plants without a previous endophyte inoculation (E−O+ plants; see red asterisks in Figure 5). Moreover, in genotypes MDV1 and VAD2, endophyte inoculation enhanced most of the JA/ET-responsive genes.

MDV1 was the genotype in which the priming effect of the endophytes was stronger. Thus, expression values in this genotype reached 130-folds in *SARD1* and *PAL1*, and 80-folds in *PR4* at 7 dpiO in E+O+, doubling the expression observed in E−O+ plants (*p* < 0.05; Figure 5 and Figure S2). Endophyte inoculation also resulted in higher expression of JA/ET-related genes compared to E−O+ plants (maximum: 3-fold change; Figure 5).

In the resistant genotype VAD2, endophyte inoculation also induced both SA and JA/ET-responsive genes after the pathogen inoculation. Regarding the SA pathway, enhanced gene expressions were observed at 7 dpiO in E+O+ compared to E−O+ plants (*p* < 0.05; Figure 5 and Figure S2). In E−O+ plants, a peak expression of SA-responsive genes was observed at 1 dpiO, and in E+O+ this overexpression was maintained (*SARD1*) or even increased (*PR4* and *WRKY70*) at 7 dpiO (Figure 5 and Figure S2). Moreover, the activation of JA/ET-responsive genes in E+O+ plants contrasts with the null activation or even repression of those genes in E−O+ plants (Figure 5). Among all the JA/ET-responsive genes evaluated, it is worth noting the strong induction of *OPR3* expression in this genotype (68.1-fold change; *p* < 0.05; Figure 5 and Figure S3). As observed in E−O+ plants of both MDV1 and VAD2, higher expression of SA-responsive genes was coupled with an overexpression of *PIP2* in E+O+ plants, with maximum expression values of a 136.4- and 75.7-fold change, respectively (Figure 5).

In contrast to the clear gene overexpression observed in E+O+ plants of MDV1 and VAD2, the modification in expression levels compared to E−O+ was much lighter in the resistant MDV2.3 genotype, for which only *PIP2* and *SARD1* genes were moderately overexpressed (Figure 5 and Figure S2).

### 3.5. Antioxidant Enzyme Activities

**Figure 6:**
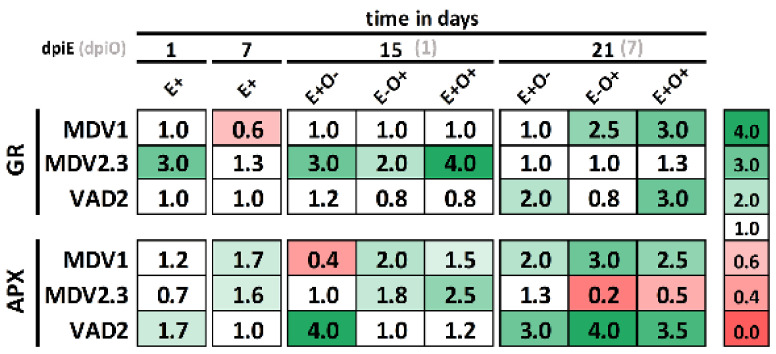
Gel activities of glutathione reductase (GR) and ascorbate peroxidase (APX) at days 1, 7, 15, and 21 of the experiment in elm genotypes MDV1, MDV2.3, and VAD2. The numbers represent the fold change relative to control plants (E−O−). Colored boxes indicate modifications of ±30% of the enzyme activity with respect to control plants, with reddish or greenish colors indicating lower or higher activity than controls, respectively.

Genotype-dependent changes were observed in the activation of the antioxidant enzymes GR and APX in response to the endophyte, *O. novo-ulmi*, or their combined inoculation. The DED-susceptible genotype MDV1 reacted to endophyte inoculation by slightly increasing APX activity and decreasing GR activity at 7 dpiE, while the resistant genotypes showed an increase in GR (MDV2.3) or APX (VAD2) at 1 dpiE (Figure 6). In MDV1, both antioxidant enzymes were activated in response to *O. novo-ulmi* inoculation at 7 dpiO, independently of the endophyte treatment. The VAD2 genotype responded to *O. novo-ulmi* (E−O+) by strongly increasing APX activity at 7 dpiO, while GR activity only increased when plants had been previously inoculated with endophytes (i.e., in E+O+ plants; Figure 6). Finally, MDV2.3 responded to *O. novo-ulmi* inoculation by increasing both GR and APX activities at 1 dpiO, this response was more intense in E+O+ plants compared to E−O+ plants (Figure 6).

## 4. Discussion

### 4.1. Different Activation of SA Genes in Response to O. novo-ulmi across U. minor Genotypes

Pathogen inoculation induced a strong activation of SA defense genes in three *U. minor* genotypes (Figure 5), in agreement with the response observed by Perdiguero et al. [57] in English elm (*Ulmus procera*). This response supports the hemibiotrophic lifestyle of *O. novo-ulmi* as proposed in previous works [70,71]: before shifting to a necrotrophic lifestyle, in which *O. novo-ulmi* produces extensive host cell death, the pathogen seems to spread as a biotroph, inducing SA biosynthesis upon plant recognition of PAMPs or pathogen effectors [8]. SA synthesis is regulated by the isochorismate and phenylpropanoid pathways [72]. These genes are usually induced after pathogen recognition and are key for SA-biosynthesis in plants [61,73]. SA accumulation commonly activates a specific defense response that implies the transcriptional reprogramming of a large number of host genes, including several WRKY transcription factors (TFs). These WRKY TFs regulate SA-responsive genes involved in fighting the pathogen such as the pathogenesis-related (PR) genes [74]. In the three elm genotypes tested, *SARD1* and *PAL1* were induced in response to *O. novo-ulmi*, suggesting SA synthesis. Nevertheless, both SA synthesis and SA responsive genes were differentially activated in each genotype.

Fast induction of SA-related genes during early pathogen invasion could be associated to higher resistance. Thus, the induction of *SARD1*, *PAL1*, and *WRKY70* at 1 dpiO, as well as their attenuation at 7 dpiO, suggests a rapid SA-dependent defense activation in the DED-resistant VAD2 genotype. On the contrary, SA-responsive genes in MDV1 were only slightly triggered at 1 dpiO and suffered a strong overexpression at 7 dpiO (Figure 5), a late response that could benefit pathogen spread. These results agree with the higher *O. novo-ulmi* presence in MDV1 than in VAD2 when pooling all stem parts (i.e., distal, intermediate, and local; *p* < 0.05). In both genotypes (MDV1 and VAD2), the overexpression of *PIP2*, a gene that is triggered in response to PAMPs and codifies for a peptide involved in amplifying plant immunity [67], was approximately proportional to the pathogen presence in xylem tissues (Figure 3). Despite MDV1 and VAD2 showing a similar induction of *PIP2* at 1 dpiO (see E−O+ plants in Figure 5), SA-mediated responses were triggered with different intensities in both genotypes, suggesting that *PIP2* recognition by receptor/like kinases (RLKs) or the resulting signal transmission for defense activation was more efficient in VAD2 than in MDV1, at least during the first hours of infection.

The higher pathogen load observed in MDV1 compared to the resistant genotypes (MDV2.3 and VAD2, Figure 3) could also be influenced by xylem vessel anatomy. Wider [54] and longer (authors, unpublished results) vessels have been observed in MDV1 than in MDV2.3 and VAD2 plants. These different anatomical features can influence the rate of pathogen entry and dispersal [75,76,77]. Wide and long vessels in MDV1, together with a delayed SA-mediated defense activation, may enhance pathogen colonization and susceptibility to *O. novo-ulmi* in this genotype.

A different behavior was detected in the resistant genotype MDV2.3. The significant *PIP2* and *SARD1* stimulation at 1 dpiO suggests pathogen recognition, which would lead to the upregulation of the SA-responsive gene *PR4* and the repression of some JA/ET-responsive genes (Figure 5). However, the small defense activation during early infection stages suggests that alternative mechanisms determine MDV2.3 resistance to DED. One such mechanism appears to be the antioxidant system activation (Figure 6). The fast, coordinated activation of GR and APX in MDV2.3, two principal enzymes of the ascorbate-glutathione (AsA-GSH) pathway, suggests high ROS levels produced by the host as a primary defense reaction against pathogen cells, an efficient recognition of ROS increment, and a rapid reaction to balance the redox state. The increase in ROS levels could act as a signal that rapidly propagates the defense activation to distal parts. In fact, ROS signaling has been described in plant responses to different stresses [9]. Moreover, small xylem vessels of MDV2.3 are also likely involved in DED resistance [54].

Plant physiology was not altered by *O. novo-ulmi* inoculation (Figure S1), supporting the previous observation that young plantlets show few DED symptoms, if any [53]. However, vascular browning occupied a larger area in the susceptible genotypes than in the resistant genotypes (Figure 2), possibly as consequence of an elevated *O. novo-ulmi* spread at 7 dpiO, as stated in other wilt diseases [78]. This result contradicts the work of Duchesne et al. [55] who reported larger browning areas in vascular tissues of resistant than of susceptible elm genotypes. Vascular browning is the consequence of different tree responses to infection, such as vessel occlusion with gels and tyloses, and accumulation of phenolics and other defense compounds in parenchyma cells [79]. In our experiment, the *PAL1* overexpression (directly involved in the synthesis of phenolic compounds) matched well with the percentage of vascular browning in each genotype. Therefore, we argue that accumulation of phenolics is not per se related to resistance, as suggested in previous works [51,71]. Rather, the rapid *PAL1* induction (as observed in VAD2 at 1 dpiO) is probably more effective than a stronger but later activation of the phenylpropanoid metabolism.

### 4.2. Fungal Endophytes Activate a Transient SA and JA Response and Act as Priming Stimulators

Besides genetic resistance, the fungal community is postulated to be an important additional defense layer contributing to elm resistance to *O. novo-ulmi*. Previous research evidenced the role of the three elm endophytes used in this study in enhancing resistance to *O. novo-ulmi* (YCB36 strain) or to abiotic stress (P5 and YM11 strains) [47,48]. Here, endophytes P5 (*Cystobasium*) and YM11 (Chaetothyriales) were detected in the stem of non-endophyte inoculated E−O− plants, supporting the idea that they belong to the core microbiome and are extensive colonizers of the stem [46], unlike YCB36 which was barely present in E−O− (Figure 4). The establishment of endophytes following artificial inoculations (E+O− plants) was genotype-dependent. While in VAD2 the abundance of P5 and YCB36 increased, in MDV1, only the presence of P5 increased. Contrary to YCB36, the load of P5 and YM11 was maintained despite *O. novo-ulmi* occurrence, indicating that they occupied a stable niche in elm tissues.

Endophyte inoculation per se stimulated a moderate SA-dependent response during the initial days of interaction with the plant in all elm genotypes (Figure 5). As observed in response to *O. novo-ulmi*, the upregulation of *PIP2* in all genotypes in response to endophyte inoculation indicates that endophytic MAMPs are recognized by *U. minor*, triggering a moderate SA-defense response. Higher overexpression of SA-dependent genes in VAD2 and MDV1, rather than in MDV2.3, seemed to be associated to the *PIP2* expression level, which in turn varied according to the endophytic load in each genotype (Figure 4). Moreover, a slight upregulation of JA/ET-dependent genes complemented the main SA-related response at 1 dpiE, suggesting that the activation of both hormone pathways occurs during endophyte colonization. In fact, *MYC2*, which is a key transcriptional regulator of JA-dependent defenses [80], was induced after endophyte inoculation in the two DED-resistant genotypes (Figure 5). The activation of the SA pathway alone or in combination with the JA/ET pathway is not commonly triggered during the colonization of beneficial microorganisms, the single activation of JA/ET being the most common response prompted by plant growth-promoting rhizobacteria and fungi (PGPR and PGPF) [21,24,81,82,83,84]. Nevertheless, in line with our results, the induction of SA-related defense genes alone or in combination with JA/ET-responsive genes has been observed in other studies with beneficial microorganisms such as *Trichoderma hamatum* [85] or *Bacillus cereus* [86].

The expression levels of SA-related genes tended to diminish at 7 dpiE (Figure 5), probably upon recognition of the non-virulent behavior of the endophytes. The slight but transient induction of host defenses after the initial recognition of the symbiotic fungi as a potential invader is a common phenomenon displayed by mycorrhiza, rhizobacteria, and other symbiotic microorganisms [30,87]. The activation of the antioxidant enzymes APX and/or GR during endophyte establishment suggests that ROS accumulates during endophyte recognition and triggers a transient induction of the plant defense in order to maintain the symbiosis [23,88,89]. The JA/ET-mediated responses also diminished at 7 dpiE but only in VAD2 plants (Figure 5). The highly effective colonization of YCB36 in VAD2, but not in MDV1 or MDV2.3, may be related with this different response. During the colonization process, microbes can adopt an active role in determining how plants react through the release of small effector proteins or small interfering RNAs to target plant transcripts and silence their translation [90]. For example, effector proteins from arbuscular mycorrhizal and ectomycorrhizal fungi have been described to interact with ET or JA receptors during the colonization process to repress their signaling pathways and enhance fungal establishment [90,91,92,93,94]. Similarly, as occurring in pathogens, the cross-kingdom exchange of small-RNAs from endophytes to the host may attenuate plant hormone signaling pathways [95].

### 4.3. A Rapid and Strong Defense Activation against O. novo-ulmi Is Triggered in Endophyte-Primed Plants

The “information” generated during the transient stimulation of SA and JA-dependent defenses after endophyte inoculation was possibly “stored” by epigenetic mechanisms until the pathogen inoculation [29,96]. Specific chromatin modifications on gene promoters, through DNA methylations or histone modifications, or accumulation of mitogen-activated protein kinases, are essential in the priming process involved in sensitizing plants for rapid and robust defense activation [97,98,99]. We clearly observed a faster and stronger defense activation upon *O. novo-ulmi* inoculation in plants previously inoculated with endophytes, which was more evident in MDV1 and VAD2 genotypes than in MDV2.3 (Figure 5). In MDV1 and VAD2, both SA and JA-mediated responses were enhanced after *O. novo-ulmi* inoculation in E+O+ compared to E−O+ plants (see red asterisks in Figure 5). Given that *O. novo-ulmi* inoculation promoted a strong but late activation of SA-dependent defenses in MDV1, the earlier SA-mediated response in endophyte-primed plants could explain the reduced *O. novo-ulmi* presence in E+O+ plants of this genotype. In MDV2.3, the endophyte-induced enhancement of APX and GR activities at 1 dpiO may indicate an endophyte-mediated increased ROS sensitivity and enhanced capacity to maintain cell homeostasis [23].

Notwithstanding the similar endophyte-induced priming response to *O. novo-ulmi* inoculation in VAD2 and MDV1, a different balance between JA/ET and SA-activation was observed. While in the susceptible MDV1, the SA-related pathway was prioritized, with a 130-fold change in some genes at 7 dpiO, the resistant VAD2 maintained an equilibrium in the stimulation of both pathways. The fast SA-response of VAD2 to *O. novo-ulmi* (described in the previous section for E−O+ plants) was not enhanced in endophyte-primed (E+O+) plants. Furthermore, while the primed expression of SA-responsive genes in VAD2 increased moderately at 7 dpiO compared to 1 dpiO, SA-related gene expression in MDV1 doubled the already elevated basal expression observed in E−O+ plants. In both cases, the SA-related response was accompanied by a JA/ET-related response, a phenomenon exclusively observed in endophyte-inoculated plants and other works using inoculations of multiple strains [100,101,102]. Regardless of the reported antagonism of SA and JA/ET-mediated defenses [6], the parallel activation of SA and JA/ET pathways [103,104] can lead to a more efficient protection against specific pathogens [7]. The moderate upregulation of JA/ET-related genes in MDV1 contrasts with the overexpression values observed in VAD2 (Figure 5), for which the high upregulation of *OPR3* suggests an increase in JA synthesis. This, however, was not related to a significant reduction of pathogen load, probably due to the basal resistance of this genotype and the low pathogen spread in E−O+ plants.

It is worth noting that the elevated activation of JA/ET-signaling during the priming in VAD2 matched well with the high establishment of the YCB36 endophyte in this genotype (Figure 4). A recent study on DED-susceptible *U. minor* trees inoculated exclusively with YCB36 revealed a reduction of *O. novo-ulmi* foliar wilting symptoms [47]. The YCB36 priming effect on JA/ET-pathways observed here could explain such lower wilting. Despite the fact that JA does not seem to be involved in the *U. minor* response to DED (Figure 5) [57], other works on *U. americana* reported a punctual activation of JA four days after the pathogen introduction [71].

## 5. Conclusions

Our study provides new evidence of the role of fungal endophytes on the priming of plant defense responses. Endophytic recognition by *U. minor* upon inoculation and the resulting transient defense stimulation mediated primarily by SA and secondarily by JA are possibly key processes in the modulation of the priming response observed when plants are exposed to *O. novo-ulmi*. The high influence of the plant genotype on both the endophyte colonization and the plant response to *O. novo-ulmi* complicates the interpretation of the priming response. The slight priming of defense genes observed in MDV2.3 was counteracted by its high and early antioxidant response, while MDV1 and VAD2 displayed a stronger priming of defense genes driven by SA and JA. The faster and stronger SA-dependent priming response observed in MDV1 was possibly involved in the reduced pathogen proliferation. This result suggests that priming could have beneficial effects on DED-susceptible genotypes. Priming stimulation by fungal endophytes opens new possibilities in the fight against DED and the management of surviving *U. minor* stands.

## Data Availability

The data presented in this study are available upon request from the corresponding author.

## Supplementary Materials

The following are available online at https://www.mdpi.com/article/10.3390/jof7090687/s1, Figure S1: Mean values (±standard error) of net photosynthesis (P_n_), stomatal conductance (g_s_), intercellular CO_2_ concentration (C_i_), and transpiration (E) in elm genotypes MDV1, MDV2.3, and VAD2, measured at day 21 of the experiment. Treatment combinations: E−O− plants (mock-inoculated plants in both treatment dates), E−O+ plants (mock-inoculated plants subsequently inoculated with *O. novo-ulmi*), E+O− plants (endophyte-inoculated plants subsequently mock-inoculated), and E+O+ plants (endophyte-inoculated plants subsequently inoculated with *O. novo-ulmi*). Different letters indicate significant differences between treatments according to Tukey’s HSD post-hoc test (*p* < 0.05). Figure S2: Mean values (±standard error) of *PIP2* and salicylic-acid responsive genes at days 1 and 7 after endophyte inoculation (E+ treatment, dpiE), and at days 1 and 7 (days 15 and 21 of the experiment) after *O. novo-ulmi* inoculation (dpiO). Treatments are: E−O− plants (mock-inoculated plants in both treatment dates), E−O+ plants (mock-inoculated plants subsequently inoculated with *O. novo-ulmi*), E+O− plants (endophyte-inoculated plants subsequently mock-inoculated), and E+O+ plants (endophyte-inoculated plants subsequently inoculated with *O. novo-ulmi*). Different letters in each sampling date indicate significant differences between treatments according to Tukey’s HSD post-hoc test (*p* < 0.05). Figure S3: Mean values (±standard error) of jasmonic-acid responsive genes at days 1 and 7 after endophyte inoculation (dpiE), and at days 1 and 7 (days 15 and 21 of the experiment) after *O. novo-ulmi* inoculation (dpiO). Treatments are: E−O− plants (mock-inoculated plants in both treatment dates), E−O+ plants (mock-inoculated plants subsequently inoculated with *O. novo-ulmi*), E+O− plants (endophyte-inoculated plants subsequently mock-inoculated), and E+O+ plants (endophyte-inoculated plants subsequently inoculated with *O. novo-ulmi*). Different letters in each sampling date indicate significant differences between treatments according to Tukey’s HSD post-hoc test (*p* < 0.05). Figure S4: Images of the in gel activities of glutathione reductase (GR) and ascorbate peroxidase (APX) at days 1, 7, 15, and 21 of the experiment in elm genotypes MDV1, MDV2.3, and VAD2. The SDS-PAGE and native gels for each sample are shown above GR and APX activities. Table S1: Primer sequences used for pathogen (*Ophiostoma novo-ulmi*) and endophyte (P5, YM11, and YCB36) quantifications. Table S2: Description of the genes selected for expression analysis. The genes evaluated are marker genes of salicylic acid (SA), jasmonic acid (JA), or ethylene (ET) pathways. The functionality of each gene is described according to TAIR (https://www.arabidopsis.org/ (accessed on 8 April 2020)). The experiment in which those genes and the isotig ID of each gene were identified are also indicated together with the primer sequences and the resulting fragment length (bp fragment). Table S3: Results of the two-way ANOVA analysis for leaf gas exchange variables, including net photosynthesis (P_n_), stomatal conductance (g_s_), intercellular CO_2_ concentration (C_i_) and transpiration (E), and vascular browning, *O. novo-ulmi* presence (in local, intermediate, and distal parts) and endophyte presence (YM11, P5, and YCB36).
